# Supplementary material for: The genome sequence of Gnaphalium uliginosum L., 1753 (Asterales: Asteraceae)
Source: Wellcome Open Res. 2026 Jun 25;11:378. [Version 1] doi: 10.12688/wellcomeopenres.26681.1 (PMC13555082; doi:10.12688/wellcomeopenres.26681.1)
Supplement: Peer review history

# Reviewer report: Reviewer response for version 1

**DOI:** 10.21956/wellcomeopenres.29382.r159473

**Published:** 2026-07-29

**Authors:** Francesco Garassino

**Affiliations:** 1 University of Zurich, Zürich, Zurich, Switzerland

Christenhusz et al. present all the relevant information on this genome assembly and annotation. The rationale and methodology are clearly and concisely presented. Assembly and annotation information is clearly presented. I particularly appreciated the inclusion of links to wet lab protocols and to the bioinformatics pipelines employed in this work.

Are sufficient details of methods and materials provided to allow replication by others?

Yes

Is the rationale for creating the dataset(s) clearly described?

Yes

Are the datasets clearly presented in a useable and accessible format?

Yes

Are the protocols appropriate and is the work technically sound?

Yes

Reviewer Expertise:

Genome biology, bioinformatics, plant ecophysiology

I confirm that I have read this submission and believe that I have an appropriate level of expertise to confirm that it is of an acceptable scientific standard.

# Reviewer report: Reviewer response for version 1

**DOI:** 10.21956/wellcomeopenres.29382.r160295

**Published:** 2026-07-22

**Authors:** Ayoob Obaid Alfalahi, Ahmed Al-Neda

**Affiliations:** 1 University of Anbar, Ramadi, Al Anbar Governorate, Iraq; 2 Field Crops, University of Anbar, Ramadi, Al Anbar Governorate, Iraq

**Review report**

 The data note entitled “The genome sequence of *Gnaphalium uliginosum* L., 1753 (Asterales: Asteraceae)” presents a genome assembly of *G. uliginosum* (marsh cudweed; Streptophyta; Magnoliopsida; Asterales; Asteraceae) in part of the Darwin Tree of Life project, which produces reference genomes for eukaryotic species found in Britain and Ireland. However, after thoroughly reviewing the manuscript, my minor concerns are outlined below:

1. The background can be improved by explaining why *G. uliginosum* genome is interesting. Furthermore, if genomes of close relatives exist, please mention them.
2. Make sure that the used keywords are relevant and effective. For example, “chromosomal” is an effective keyword and can be replaced. Also, “genome assembly” is more accurate as you are presenting an assembled genome.
3. The version of the used software should be included (Hifiasm, YaHS …. etc.).
4. It would be better to specify the used barcode(s).
5. The authors mentioned that the evaluation of both the primary and alternate haplotypes was conducted using MerquryFK, but it is not clear whether the alternate haplotype assembly has been deposited and is publicly available. Please clarify this in the manuscript and provide the corresponding accession(s).
6. You may add a brief explanation for the reasonable difference between GenomeScope and flow cytometry.
7. Try not to alternate between different terms because it will be confusing (e.g. RNA sequencing and RNA-seq; HiFi reads and Pacific Biosciences single-molecule HiFi long reads).

**I wish authors would be able to use these comments to improve their article before the next submission.**

Are sufficient details of methods and materials provided to allow replication by others?

Partly

Is the rationale for creating the dataset(s) clearly described?

Partly

Are the datasets clearly presented in a useable and accessible format?

Yes

Are the protocols appropriate and is the work technically sound?

Yes

Reviewer Expertise:

My area of research is Plant Biotechnology

We confirm that we have read this submission and believe that we have an appropriate level of expertise to confirm that it is of an acceptable scientific standard.
